# Efficacy of hyperbaric oxygen therapy in postoperative patients with cerebral aneurysms: a systematic review and meta-analysis

**DOI:** 10.3389/fneur.2025.1645028

**Published:** 2025-09-11

**Authors:** Yue Gao, Xiaoyuan Sun, Guozhong Wang

**Affiliations:** ^1^Department of Hyperbaric Oxygen, Civil Aviation General Hospital, Beijing, China; ^2^Department of Gynaecology and Obstetrics, Civil Aviation General Hospital, Beijing, China

**Keywords:** intracranial aneurysm, postoperative recovery, neurological function, meta-analysis, hyperbaric oxygen therapy

## Abstract

**Background:**

Surgical management of intracranial aneurysms frequently results in postoperative neurological impairments and diminished quality of life. Hyperbaric Oxygen Therapy (HBOT) has emerged as a potential adjunctive treatment to enhance neurological recovery and functional outcomes; however, its effectiveness remains debated.

**Objective:**

This systematic review and meta-analysis aimed to assess the efficacy of HBOT on clinical outcomes, neurological improvement, functional independence, and health-related quality of life in postoperative intracranial aneurysm patients.

**Methods:**

Randomized controlled trials (RCTs) comparing HBOT combined with routine postoperative care versus routine care alone were identified through comprehensive database searches of PubMed, Web of Science, CNKI, Wanfang, and VIP up to April 2025. Data were analyzed using fixed- or random-effects models based on heterogeneity. Risk ratios (RR) and standardized mean differences (SMD), with corresponding 95% confidence intervals (CI), were calculated as summary measures.

**Results:**

A total of 11 RCTs, encompassing 2,268 patients, were analyzed. HBOT significantly improved clinical treatment efficacy (RR = 1.19, 95% CI: 1.11–1.28, *p* < 0.00001) and neurological function (SMD = −0.63, 95% CI: −0.78 to −0.48, *p* < 0.00001). Functional independence also improved markedly, demonstrated by higher scores in Activities of Daily Living (ADL; SMD = 1.24), Barthel Index (SMD = 1.00), and SF-36 total scores (SMD = 1.32). All outcomes showed statistically significant improvements with minimal heterogeneity (*I*^2^ = 0%). Sensitivity analysis confirmed the robustness of the results.

**Conclusion:**

Adjunctive hyperbaric oxygen therapy significantly enhances neurological recovery, functional outcomes, and overall quality of life in patients following intracranial aneurysm surgery. These results advocate for the clinical adoption of HBOT; nevertheless, additional high-quality, multicenter studies are necessary to confirm sustained long-term effects.

**Systematic review registration:**

https://www.crd.york.ac.uk/PROSPERO/view/CRD420251086811.

## 1 Introduction

Cerebral aneurysms, characterized by focal dilatations of intracranial arteries, represent a potentially fatal vascular disorder due to their propensity to rupture, often resulting in subarachnoid hemorrhage (SAH) (1, 2). SAH accounts for a substantial proportion of hemorrhagic strokes and is associated with high rates of morbidity and mortality, particularly in cases involving ruptured aneurysms (3). To mitigate the risk of rebleeding and improve long-term neurological prognosis, surgical interventions such as microsurgical clipping and endovascular coiling are routinely employed. Despite the efficacy of these techniques in excluding aneurysms from the cerebral circulation, postoperative outcomes are frequently compromised by a range of complications, including cerebral vasospasm, delayed cerebral ischemia (DCI), hydrocephalus, and cognitive impairment (4, 5). These sequelae not only prolong hospitalization and rehabilitation but also impose significant burdens on patients, families, and healthcare systems.

Considering these challenges, there has been increasing interest in identifying adjunctive therapeutic modalities that can enhance cerebral perfusion, limit secondary neuronal injury, and ultimately improve functional recovery. Among these, hyperbaric oxygen therapy (HBOT) has emerged as a promising candidate. HBOT involves the inhalation of 100% oxygen under elevated atmospheric pressure, typically within a sealed hyperbaric chamber (6–8). This therapeutic approach is hypothesized to exert multiple neuroprotective effects, including increased oxygen delivery to hypoxic brain tissue, attenuation of cerebral edema, reduction of oxidative stress, and modulation of neuroinflammatory pathways (9). Moreover, HBOT has been reported to promote angiogenesis and neurogenesis, thereby potentially facilitating tissue repair and functional restoration in injured brain regions.

While the neurophysiological rationale for HBOT is well-supported by experimental data and its efficacy has been explored in several neurological conditions, most notably ischemic stroke and traumatic brain injuries, its clinical utility in the context of postoperative management of cerebral aneurysms remains inadequately defined. A limited number of studies have investigated the role of HBOT in this population, with some suggesting potential improvements in neurological function, reduction in vasospasm incidence, and enhanced recovery (10). However, the existing body of literature is marked by heterogeneity in study design, patient populations, HBOT protocols, and outcome measures, which have hindered the derivation of definitive conclusions. Furthermore, the absence of a comprehensive and methodologically rigorous synthesis of available evidence underscores the need for further investigation.

Therefore, the present study aims to systematically evaluate the efficacy and safety of hyperbaric oxygen therapy in patients undergoing surgical treatment for cerebral aneurysms through a systematic review and meta-analysis of relevant clinical studies. By integrating data across multiple studies, this analysis seeks to quantify the impact of HBOT on key clinical outcomes, including neurological function, postoperative complications, and overall recovery trajectories. The findings of this review are intended to inform clinical decision-making and provide a foundation for future research into optimizing perioperative care in patients with cerebral aneurysms.

## 2 Methods

This systematic review and meta-analysis were conducted in accordance with the Preferred Reporting Items for Systematic Reviews and Meta-Analyses (PRISMA) guidelines. A structured approach was adopted to ensure methodological rigor in the identification, selection, evaluation, and synthesis of relevant clinical studies on the efficacy of hyperbaric oxygen therapy (HBOT) in patients following surgical treatment for cerebral aneurysms.

### 2.1 Eligibility criteria

Studies were included in this review based on predefined inclusion and exclusion criteria. Eligible studies comprised randomized controlled trials (RCTs), prospective cohort studies, retrospective observational studies, and case–control studies that evaluated the effects of HBOT in postoperative patients with cerebral aneurysms. Participants were required to be adult patients (aged 18 years or older) who had undergone surgical treatment for intracranial aneurysms, including microsurgical clipping or endovascular coiling. The intervention of interest was hyperbaric oxygen therapy, defined as the administration of 100% oxygen at a pressure greater than atmospheric pressure (usually >1.4 ATA), delivered in a hyperbaric chamber. The comparison group consisted of patients who received standard postoperative care with or without sham therapy, but without exposure to HBOT. Eligible studies were required to report at least one relevant clinical outcome. Studies were excluded if they were case reports, narrative reviews, editorials, animal or *in vitro* studies, or lacked a control group.

### 2.2 Information sources

A comprehensive search of multiple electronic databases was conducted to identify all relevant studies published up to 15 May 2025. The databases searched included PubMed, Web of Science, the China National Knowledge Infrastructure (CNKI), and Wanfang and VIP Data. To enhance completeness, we also manually reviewed the reference lists of all included articles and relevant reviews to identify additional eligible studies.

### 2.3 Search strategy

The search strategy was designed to maximize sensitivity and specificity by combining both medical subject headings (MeSH) and free-text keywords related to cerebral aneurysms, surgical intervention, and hyperbaric oxygen therapy. Boolean operators (“AND,” “OR”) were used to link search terms, and the strategy was customized for each database. An example of the PubMed search string was: (“cerebral aneurysm” OR “intracranial aneurysm”) AND (“surgery” OR “clipping” OR “coiling”) AND (“hyperbaric oxygen” OR “HBOT”). Searches were limited to human studies published in English or Chinese.

### 2.4 Study selection process

**Figure 1:**
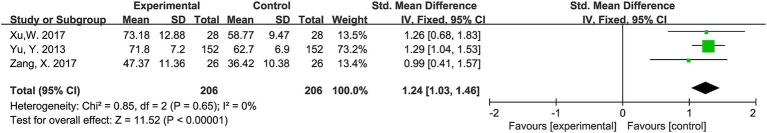
PRISMA flow diagram of study selection.

All identified records were imported into EndNote X9 (Clarivate Analytics) for de-duplication. Two independent reviewers conducted an initial screening of titles and abstracts to exclude studies that clearly did not meet inclusion criteria. The full texts of potentially eligible studies were then retrieved and assessed independently by the same reviewers. Disagreements regarding inclusion were resolved through discussion, and a third reviewer was consulted in cases of persistent disagreement. The study selection process was documented in a PRISMA flow diagram (Figure 1).

### 2.5 Data extraction and management

Data from included studies were extracted independently by two reviewers using a predesigned, standardized data extraction form created in Microsoft Excel. The extracted information included the following: first author, year of publication, country, study design, sample size, patient characteristics (age and sex), follow-up duration and outcomes. Discrepancies in data extraction were resolved through discussion, with involvement of a third reviewer when necessary.

### 2.6 Risk of bias assessment

The methodological quality of each included study was assessed independently by two reviewers. For randomized controlled trials, the Cochrane Risk of Bias tool (RoB 1.0) was used to evaluate bias across five domains: the randomization process, deviations from intended interventions, missing outcome data, measurement of the outcome, and selection of the reported result.

### 2.7 Data synthesis and statistical analysis

Meta-analyses were performed for outcomes reported by at least two studies using Review Manager (RevMan, version 5.4). For dichotomous outcomes, risk ratios (RRs) with 95% confidence intervals (CIs) were calculated. For continuous outcomes, standardized mean differences (SMDs) were used, depending on the consistency of measurement scales across studies. Statistical heterogeneity was assessed using the chi-square (Q) test and quantified with the *I*^2^ statistic. And *I*^2^ value above 50% was considered indicative of substantial heterogeneity. A random-effects model was applied in the presence of moderate to high heterogeneity; otherwise, a fixed-effect model was used. Sensitivity analyses were performed sequentially excluding individual studies to assess the stability of the pooled estimates.

## 3 Results

### 3.1 Study selection

A total of 450 records were initially retrieved from six electronic databases: PubMed, Web of Science, CNKI, Wanfang, and VIP. After removing 39 duplicate records, 411 articles remained for screening. After reading the titles and abstracts, 363 articles were excluded due to irrelevance. The remaining 48 articles were sought for full-text retrieval. One study was excluded because the full text was unavailable. A total of 47 full-text articles were assessed for eligibility. Among these, 34 were excluded because hyperbaric oxygen therapy was combined with other interventions, and 3 were excluded due to insufficient extractable data. Finally, 11 studies (11–21) met the inclusion criteria and were included in the meta-analysis.

### 3.2 Study characteristics

A total of 11 randomized controlled trials (RCTs) were included in this meta-analysis, with publication years ranging from 2007 to 2025. The total sample size was 1,132 patients in the experimental group and 1,136 in the control group. All included studies were conducted in China and focused on the clinical efficacy of hyperbaric oxygen therapy following surgery for intracranial aneurysms. Detailed study characteristics are summarized in Table 1.

**Table 1:** Characteristics of the included studies in the meta-analysis.

Author	publish year	Article type	Sample size (E/C)	Sexual ratio, E	Sexual ratio, C	Age, y, E	Age, y, C	Follow-up	Research period
Chen (11)	2012	RCT	152/152	64/88	68/54	53.4 ± 6.1	53.2 ± 6.2	6 months	2004.1 to 2010.12
Cui (12)	2025	RCT	50/20	32/18	30/20	56.18 ± 5.22	55.39 ± 5.41	12 months	2020.1 to 2022.5
Hou (13)	2020	RCT	62/62	32/30	33/29	53.9 ± 2.1	54.3 ± 1.8	6 months	2018.1 to 2019.5
Jiang (14)	2014	RCT	100/100	95/105		47.5±/		6 months	2009.12 to 2013.12
Kuang (15)	2007	RCT	50/50	23/27	21/29	48.2±/		1 month	1996.1 to 2006.1
Ma (16)	2022	RCT	150/150	147/153		47.4 ± 13.5		1 month	2018.11 to 2020.11
Xiao (17)	2014	RCT	41/48	25/16	29/19	>18	>18	1 month	2008.1 to 2013.12
Xu (18)	2017	RCT	28/28	18/10	10/18	41.25 ± 5.8	41.84 ± 5.6	1 month	2014.4 to 2015.4
Yu(19)	2018	RCT	50/50	28/22	30/20	41.52 ± 5.01	41.21 ± 5.25	3 months	2017.2 to 2018.2
Yu (20)	2013	RCT	150/150	64/88	68/54	53.2 ± 6.2	53.4 ± 6.1	6 months	2004.1 to 2010.12
Zang et al. (21)	2017	RCT	26/26	12/14	13/13	45.4 ± 13.8	45.2 ± 13.7	6 months	2016.1 to 2017.1

### 3.3 Risk of bias within studies

**Figure 2:**
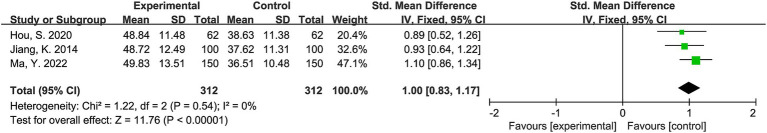
Summary of risk of bias for each domain across all included studies.

**Figure 3:**
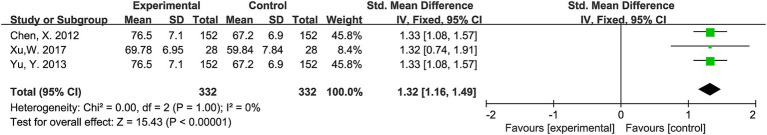
Risk of bias assessment for each included study.

The risk of bias in the included randomized controlled trials was evaluated using the Cochrane Risk of Bias tool, as shown in Figures 2, 3. Most studies had a low risk of bias for random sequence generation. However, allocation concealment and blinding of participants and personnel frequently showed unclear or high risk, indicating potential selection and performance bias. About half of the studies had unclear or high risk for blinding of outcome assessment, suggesting possible detection bias. In contrast, most studies showed a low risk for incomplete outcome data and selective reporting, reflecting generally adequate data quality. Overall, the methodological quality was moderate, with limited reporting on allocation and blinding procedures in several studies.

### 3.4 Synthesis of results

#### 3.4.1 Treatment effectiveness rate

**Figure 4:**
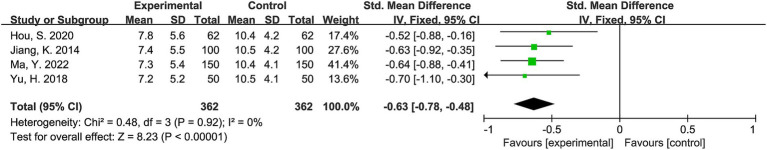
Forest plot of treatment effectiveness rate comparing hyperbaric oxygen therapy with control.

A total of four studies reported data on treatment effectiveness rate. The pooled analysis showed that the HBO group had a significantly higher effectiveness rate compared to the control group, with a combined RR of 1.19 (95% CI: 1.11–1.28, *p* < 0.00001), indicating a 19% improvement in treatment response. There was no significant heterogeneity among studies (*I*^2^ = 0%, *p* = 0.96), so a fixed-effects model was used (Figure 4). These results suggest that HBO therapy significantly improves clinical outcomes in postoperative patients with intracranial aneurysms.

#### 3.4.2 Neurological function deficit (NFD)

**Figure 5:**
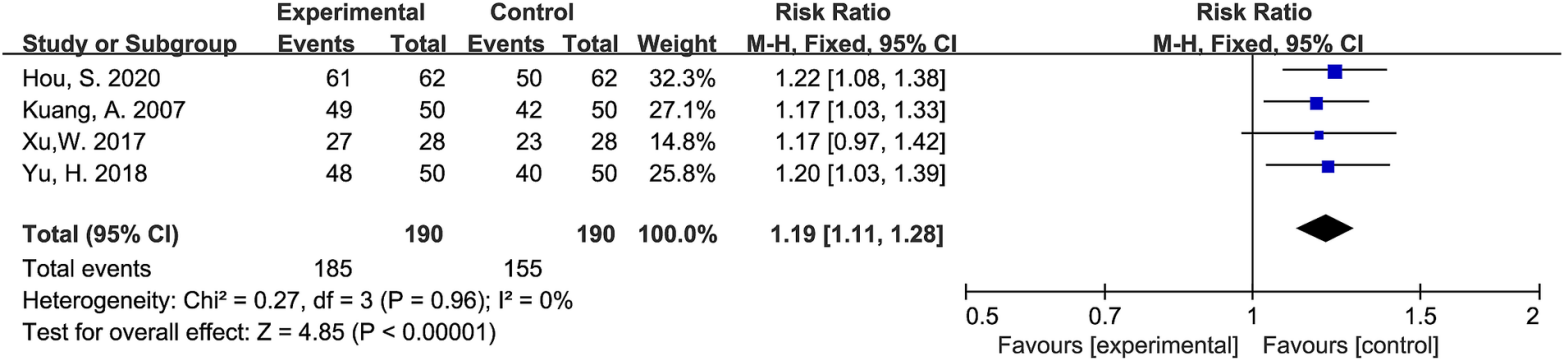
Forest plot of neurological function deficit (NFD) scores comparing hyperbaric oxygen therapy with control.

Four studies reported data on NFD scores. A fixed-effects model was applied due to low heterogeneity among studies (*I*^2^ = 0%, *p* = 0.92). The pooled SMD was −0.63 (95% CI: −0.78 to −0.48, *p* < 0.00001) (Figure 5), indicating a statistically significant improvement in neurological function in the hyperbaric oxygen therapy group compared to the control group. These results suggest that HBO therapy is effective in reducing neurological deficits after surgery for intracranial aneurysms.

#### 3.4.3 Activities of daily living (ADL) score

**Figure 6:**
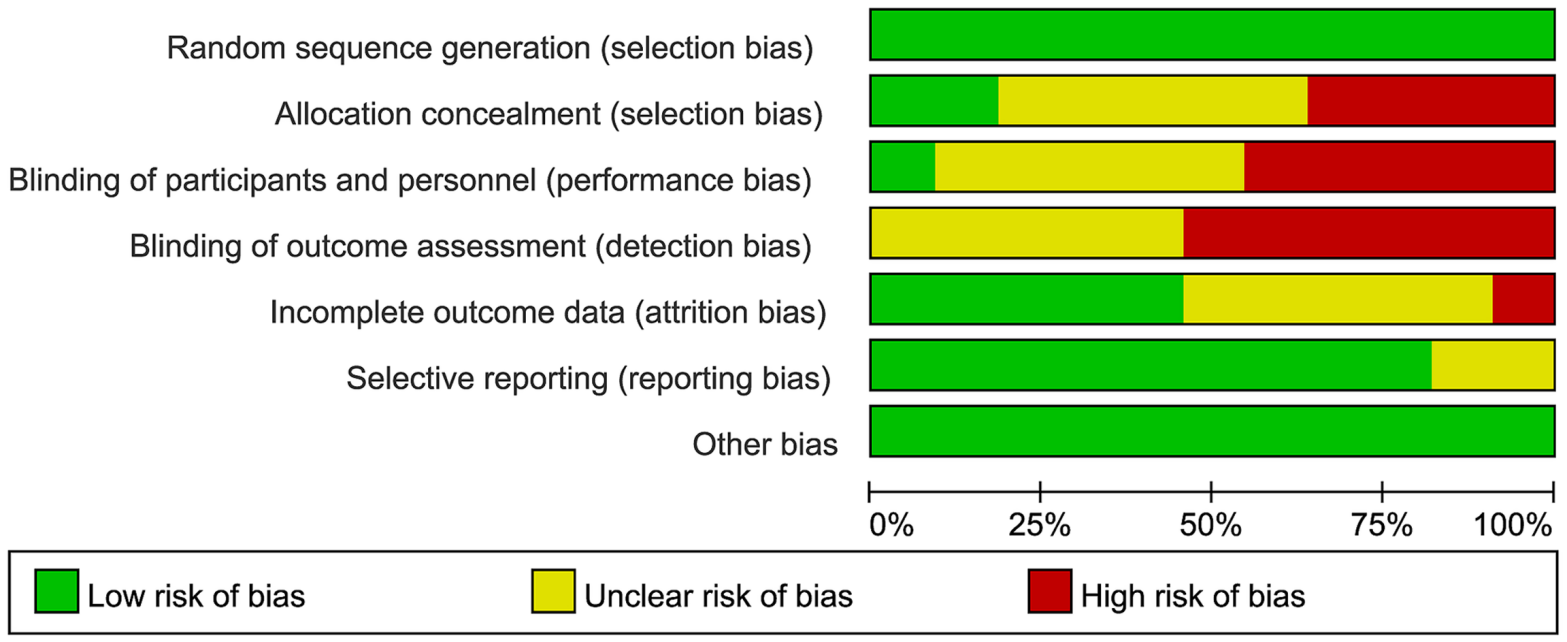
Forest plot of ADL scores comparing hyperbaric oxygen therapy with control.

Three studies reported ADL scores. A fixed-effects model was used due to low heterogeneity (*I*^2^ = 0%, *p* = 0.65). The pooled SMD was 1.24 (95% CI: 1.03–1.46, *p* < 0.00001) (Figure 6), indicating that hyperbaric oxygen therapy significantly improved patients’ functional independence compared to the control group. These results demonstrate that HBO therapy can effectively enhance the postoperative activities of daily life in patients with intracranial aneurysms.

#### 3.4.4 Barthel Index

**Figure 7:**
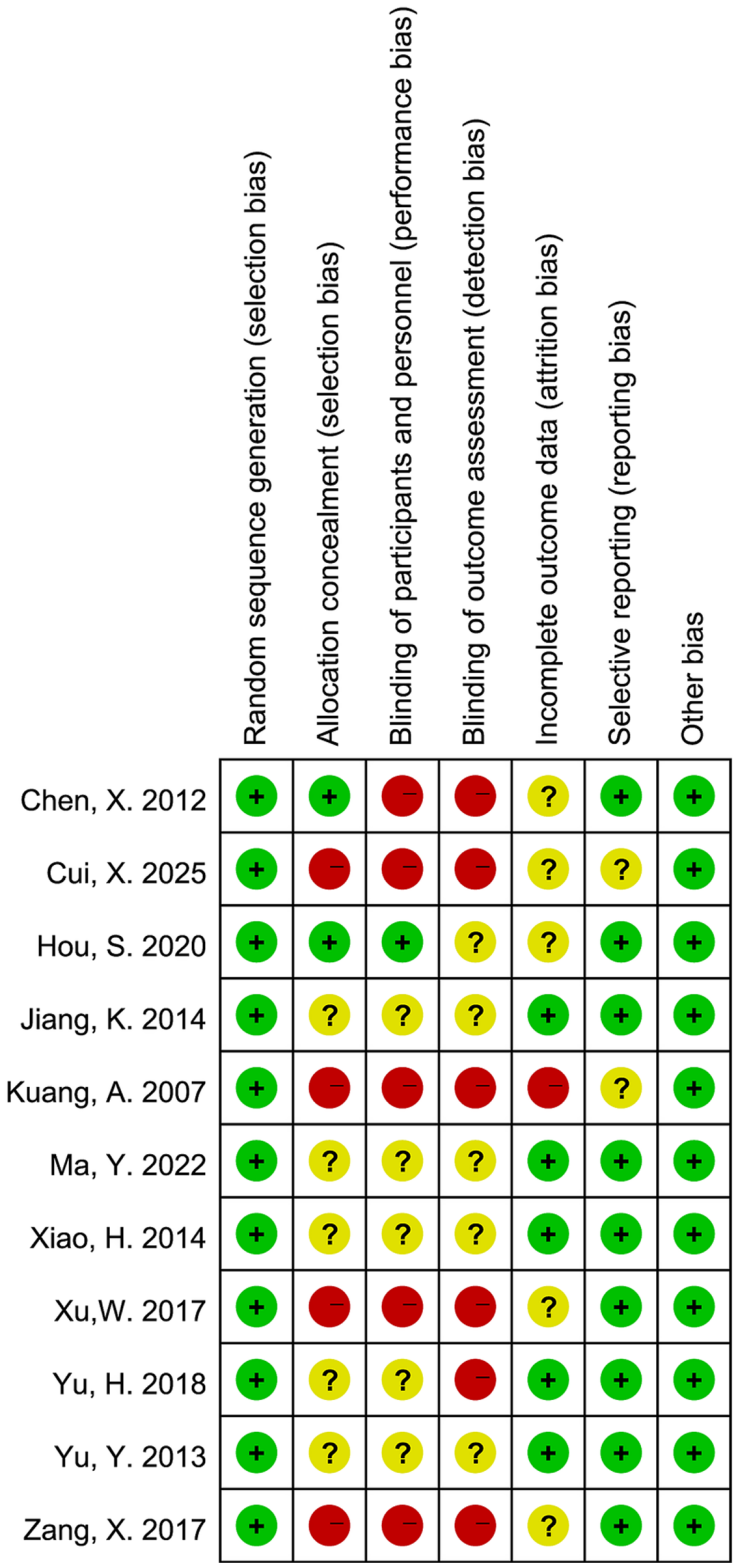
Forest plot of Barthel Index scores comparing hyperbaric oxygen therapy with control.

Three studies reported Barthel Index scores to assess daily living ability. Due to the absence of heterogeneity (*I*^2^ = 0%, *p* = 0.54), a fixed-effects model was applied. The pooled SMD was 1.00 (95% CI: 0.83–1.17, *p* < 0.00001) (Figure 7), indicating a significant improvement in functional independence in the hyperbaric oxygen therapy group compared to the control group. These findings suggest that hyperbaric oxygen therapy effectively enhances postoperative self-care ability in patients with intracranial aneurysms.

#### 3.4.5 SF-36 total score

**Figure 8:**
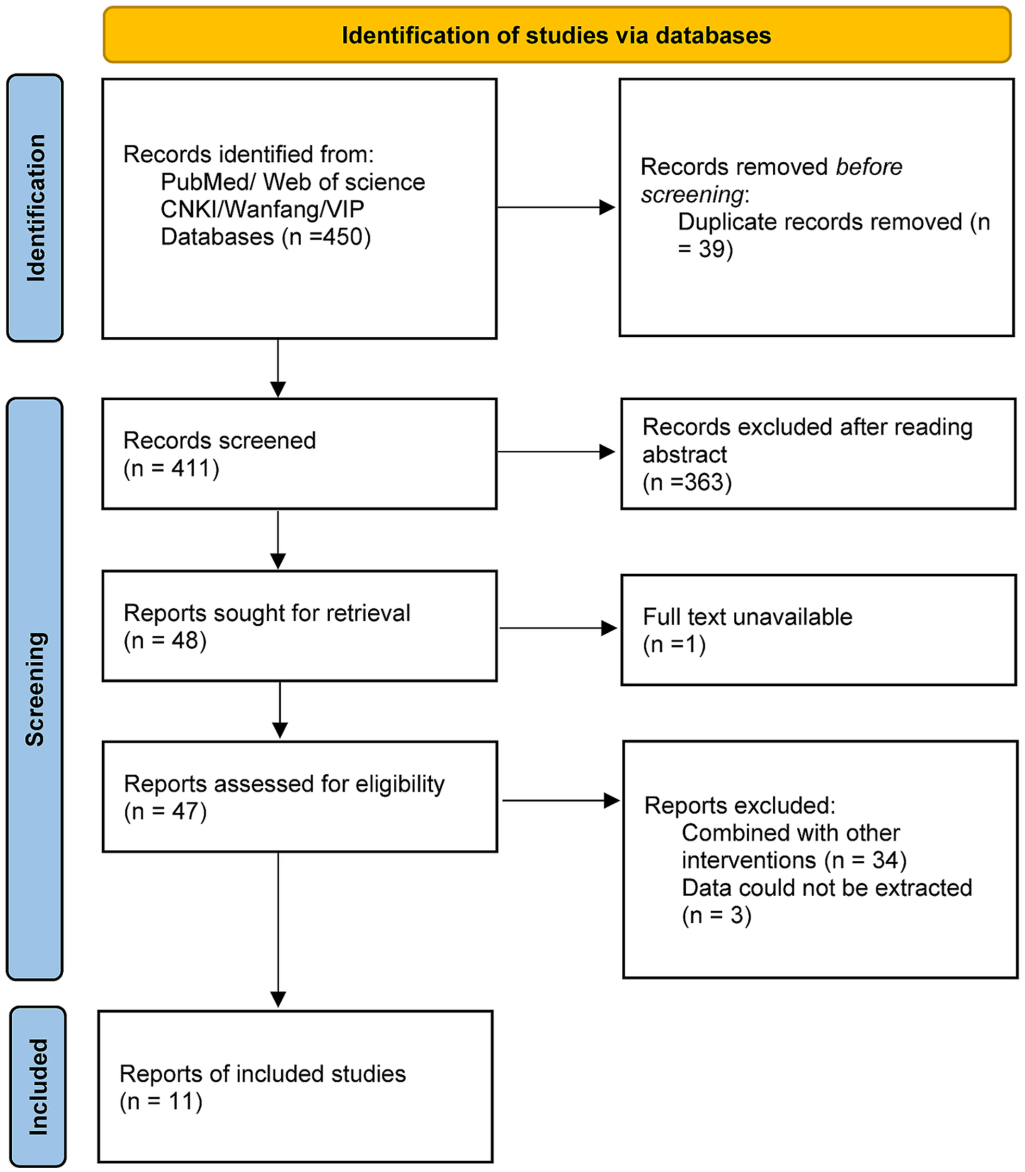
Forest plot of SF-36 total scores comparing hyperbaric oxygen therapy with control.

Three studies reported SF-36 total scores to assess overall quality of life. A fixed-effects model was used due to no heterogeneity across studies (*I*^2^ = 0%, *p* = 1.00). The pooled SMD was 1.32 (95% CI, 1.16–1.49, *p* < 0.00001) (Figure 8), indicating a significant improvement in health-related quality of life in the hyperbaric oxygen therapy group compared to the control group. These findings suggest that HBO therapy significantly enhances postoperative quality of life in patients with intracranial aneurysms.

### 3.5 Sensitivity analysis

To assess the robustness of the meta-analysis results, sensitivity analysis was conducted by sequentially removing each included study and reanalyzing the pooled estimates. The results showed that the overall effect sizes did not change substantially after excluding any single study, indicating that the findings are stable and not overly influenced by any individual study. In addition, the use of fixed- and random-effects models yielded consistent results, further supporting the reliability of the analysis.

## 4 Discussion

This meta-analysis provides comprehensive evidence on the clinical benefits of hyperbaric oxygen therapy (HBOT) in patients undergoing surgery for intracranial aneurysms. By synthesizing data from 11 randomized controlled trials, we found that HBOT significantly improves a range of postoperative outcomes, including treatment response, neurological function, functional independence, and health-related quality of life.

The pooled analysis showed that HBOT is associated with a significantly higher treatment effectiveness rate compared to conventional care. This finding suggests that the addition of HBOT may enhance postoperative recovery, possibly by improving oxygen delivery to the brain and accelerating the resolution of ischemia-induced tissue damage. Moreover, neurological function, as assessed by standardized deficit scores, was markedly better in the HBOT group (12–14), indicating that oxygen therapy may play a key role in neural repair processes following aneurysmal surgery. Functional status, measured through ADL and Barthel Index scores, also favored the HBOT group. These results are clinically meaningful, as they demonstrate improved self-care and daily function—critical components of patient recovery and reintegration into normal life (15, 16). In addition, quality-of-life outcomes assessed by the SF-36 questionnaire revealed significant improvements in patients receiving HBOT, emphasizing the broader physical and psychological benefits of this intervention.

Increased partial pressure of oxygen facilitates oxygen diffusion into hypoxic tissue, supports mitochondrial function, and improves cellular metabolism (22). HBOT has also been shown to reduce cerebral edema, suppress inflammation, modulate oxidative stress, and promote angiogenesis and neurogenesis—all of which contribute to better neurological and functional outcomes after brain injury or surgery (13, 23). These mechanisms provide a strong physiological basis for the observed clinical benefits. Importantly, the results of this analysis demonstrated high consistency across studies, with low statistical heterogeneity for all outcomes. Sensitivity analyses confirmed that the pooled estimates were stable and not significantly influenced by any single study, which enhances confidence in the robustness of the findings. The convergence of effect sizes across multiple domain clinical efficacy, function, and quality of life—further reinforces the reliability of the evidence.

Despite these strengths, several limitations warrant consideration. The methodological quality of the included studies was moderate; although randomization was reported in all trials, details regarding allocation concealment and blinding were often insufficient or unclear, raising potential concerns of selection and performance bias. Additionally, all studies were conducted in China, which may limit generalizability to other healthcare settings, populations, or ethnic groups. Sample sizes in some trials were relatively small, and most studies only reported short-term outcomes, making it difficult to assess the long-term effectiveness and safety of HBOT in this context. Furthermore, variations in outcome measurement tools—especially for functional and quality-of-life indices—may introduce reporting variability, even though statistical heterogeneity remained low. Future research should address these limitations by conducting large-scale, multicenter randomized controlled trials with rigorous methodology, standardized outcome measures, and extended follow-up periods. It would also be valuable to explore cost-effectiveness, safety profiles, and patient-centered outcomes to better inform clinical practice and policy decisions.

## 5 Conclusion

In summary, this meta-analysis supports the use of hyperbaric oxygen therapy as an effective adjunct to standard postoperative management in patients with intracranial aneurysms. HBOT appears to enhance neurological recovery, functional rehabilitation, and quality of life without introducing significant heterogeneity or instability in the evidence. These findings provide a strong rationale for integrating HBOT into perioperative care protocols, although further high-quality studies are needed to confirm and extend these results.

## Data Availability

The raw data supporting the conclusions of this article will be made available by the authors, without undue reservation.
